# Non-Ketotic Hyperglycemic Hemichorea-Hemiballismus in the Setting of Antipsychotics and Methamphetamine

**DOI:** 10.7759/cureus.19094

**Published:** 2021-10-28

**Authors:** Colbert C Nelson, Cole Ohnoutka, Michael Ulen

**Affiliations:** 1 Family and Community Medicine, University of Missouri, Columbia, USA

**Keywords:** chorea hyperglycemia basal ganglia syndrome, anti-psychotic, bipolar, amphetamine abuse, hyperglycemic hemichorea, nonketotic hyperglycemia

## Abstract

The management of common chronic conditions presenting in uncommon ways is an important facet of medical education and patient care. We report a 54-year-old patient who developed left arm dystonia precipitated by several potential factors. These include laboratory findings notable for significant hyperglycemia, methamphetamine positive on urine drug testing and patient history consistent with bipolar II managed with Lurasidone, a second-generation antipsychotic medication. The potential etiology of this uncommon presentation is discussed below.

## Introduction

Chorea is an involuntary movement disorder that affects the peripheral limbs, neck or face that typically move from different areas of the body. The primary cause of chorea is thought to be associated with imbalances of dopamine in relation to the basal ganglia. Chorea can be secondary to many diagnoses including neurological disorders, metabolic disorders, autoimmune disorders, pharmacological agents; both illicit and prescribed [1]. This case report focuses on a case of chorea secondary to uncontrolled hyperglycemia, resulting in non-ketotic hyperglycemic hemichorea-hemiballismus. Hemichorea can result from pharmacological agents, in this case, both second-generation antipsychotics and methamphetamine are present and are potential contributors to our patient’s presentation. In this case report, we describe a non-ketotic hyperglycemic hemichorea-hemiballismus with other potential secondary etiologies also present [1-16].

## Case presentation

A 54-year-old female was admitted to the hospital due to generalized left arm restlessness and pain. On the day of admission, patient presented to the emergency department with concerns of involuntary neck and left arm movement, left arm and neck pain and dizziness. On initial examination, vitals were notable for temperature of 36.8 °C, blood pressure of 196/122 mmHg, heart rate 107 beats per minute, respiratory rate 20 breaths per minute, oxygen saturation 100% on room air. She was alert, oriented and cooperative during the examination. Patient appeared restless and anxious with left-sided facial grimacing, involuntary left arm non-rhythmic jerking and left-sided neck spasm. Mild agitation throughout due to pain and discomfort. Left upper and lower extremity examination limited secondary to involuntary jerking; however, no rigidity or spasticity noted, 4+/5 strength in left-hand grip strength. No abnormal movements in the right upper and lower extremity, 5/5 strength on right side. No focal neurological deficits were observed with cranial nerves 2-12 grossly intact. Patient's symptoms were unable to be distracted while awake; however, movements would resolve with sleep.

Laboratory analysis in the ED revealed an unremarkable complete blood count (CBC), complete metabolic panel (CMP) significant for mild hyponatremia (130 mmol/L), hypokalemia (3.4 mmol/L), and hyperglycemia (416 mg/dL). Urine drug screen (UDS) was positive for amphetamine. While in the ED patient received a total of 2L normal saline, 75mg diphenhydramine (Benadryl), 5mg diazepam (Valium) and 50mcg fentanyl which resulted in mild improvement of symptoms. Patient was admitted for management of her left arm akathisia as well as for hyperglycemia and hypertensive urgency.

**Figure 1:**
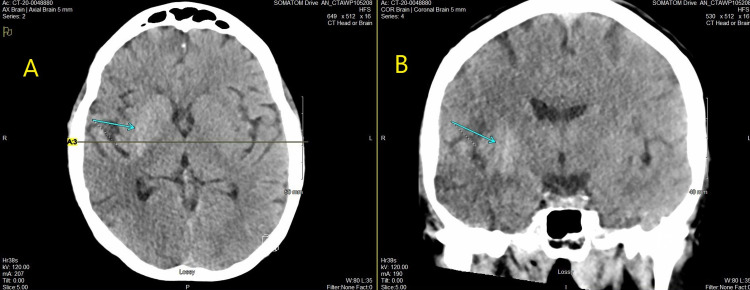
CT non-contrast scan demonstrating abnormal attenuation. (A) axial view with arrow indicating attenuation. (B) coronal view with arrow indicating attenuation. (A) Axial computed tomography (CT) non-contrast scan indicating abnormal attenuation. (B) Coronal computed tomography (CT) non-contrast scan indicating abnormal attenuation.

One month prior to admission, patient was hospitalized for somnolence, found to have a blood glucose level above 700 mg/dL and an glycated hemoglobin (A1C) of 18%. During evaluation, computed tomography (CT) scan of her head demonstrated asymmetric decreased attenuation of the right cerebral hemisphere, predominately the right main cerebral artery (MCA) distribution along with subtle loss of gray-white matter differentiation as seen in Figure 1A, 1B. 

**Figure 2:**
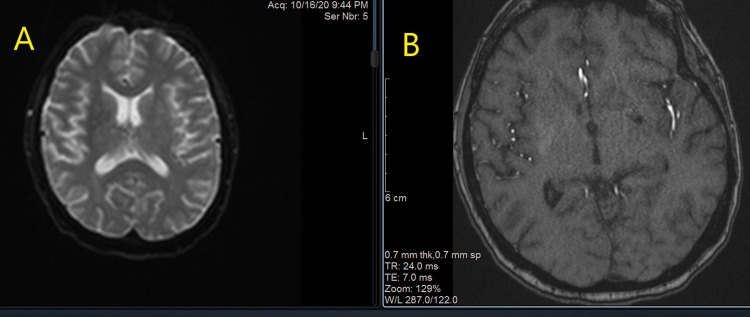
MRI (A) and MRA (B). MRI: magnetic resonance imaging; MRA: magnetic resonance angiography.

Subsequent magnetic resonance imaging (MRI) and magnetic resonance angiography (MRA) of the head and neck found no evidence of acute or subacute infarction as Figure 2A (MRI) and Figure 2B (MRA).

Somnolence and blood glucose levels were improved with intravenous fluids. Due to urinalysis positive for nitrites and chest x-ray concerning for right lower lobe pneumonia patient was treated with levofloxacin, discharged two days later with improvement of symptoms.

Two weeks prior to admission, the patient had home lurasidone dosage changed from 20mg to 40mg for management of bipolar II by primary physician. One day prior to ED presentation detailed above, patient was seen at an outside emergency department (ED) for acute onset of left upper extremity restlessness, initially treated with diphenhydramine and lorazepam. The symptoms resolved shortly after, but returned the following afternoon.

Patient has a past medical history of coronary artery disease with a left anterior descending artery stent placement 6 years prior, bipolar II, depression, hypertension, rheumatoid arthritis, diverticulitis status post colon partial resection, type 2 diabetes mellitus and methamphetamine use disorder. Home medications include insulin glargine (Lantus) 20U, metformin ) 1000mg, gabapentin 300mg three times daily, lisinopril 20mg, metoprolol tartrate 25mg bid and lurasidone 40mg.

Differential for her left arm involuntary movement included drug-induced (amphetamine/methamphetamine), vascular insult (related to imaging one month prior), extrapyramidal side effects from antipsychotic medication and conversion disorder (history of post-traumatic stress disorder (PTSD)). Upon admission, lurasidone was discontinued and Psychiatry was consulted due to left upper and lower extremity choreiform-like movements. Oral benztropine 1 milligram twice daily for abnormal movements was started along with an MRA and neurology consultation. Patient did not show improvement with discontinuation of lurasidone. Subsequently, it was restarted along with trial of 1mg intravenous haloperidol in the hopes of suppressing involuntary movements via dopamine type 2 blockade.

**Figure 3:**
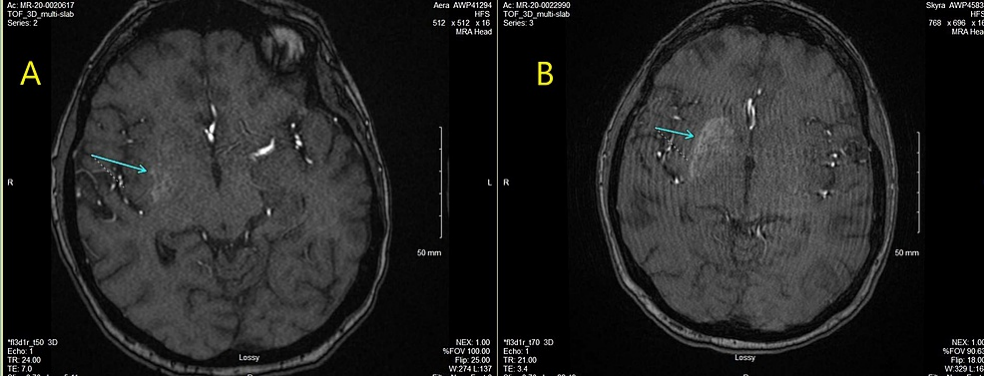
MRA brain demonstrating hyperintense signaling. (A) MRA scan during previous hospitalization one month prior; (B) one month later during current hospitalization. (A) Magnetic resonance angiography indicating hyperintense signaling during prior hospitalization one month prior. (B) Magnetic resonance angiography indicating hyperintense signaling during current hospitalization.

MRA imaging seen in Figure 3B indicated unilateral right basal ganglia hyperintense signal primarily along with the putamen portion of the caudate consistent with previous imaging (Figure 3A) from last admission but not as strong as one month prior. 

These image findings along with patient’s symptoms of choreiform like movement and persistent chronic uncontrolled hyperglycemia suggested a possible chronic cause. As the patient's symptoms were not likely extrapyramidal secondary to antipsychotic use, decision was made to continue lurasidone, bridging with haloperidol until lurasidone was therapeutic. Incrementally increased insulin regimen to 40U lantus for tighter glucose control which would most likely improve patient's symptoms. Patient contributed symptoms to lurasidone, not uncontrolled hyperglycemia so refused restarting lurasidone. Decision was made to use quetiapine and titrate to a max dose of 150 mg daily. Metoprolol was discontinued and carvedilol 6.25mg bid was added for more effective blood pressure control. Patient was discharged after nine-day hospital stay with scheduled follow-up. On discharge, patient’s symptoms including pain and dystonia were greatly improved but not completely resolved. At follow up dystonia symptoms completely resolved, however pain persisted. Patient was lost after initial follow-up status post-hospital discharge.

## Discussion

There were three main considerations for the etiology of our patient’s dyskinetic chorea-like movement. Secondary to her methamphetamine use which may have resulted in a hyperdopaminergic state Due to bipolar dopamine antagonist medication leading to extrapyramidal side effects. Lastly, dysfunction of the patient's basal ganglia secondary to uncontrolled severe hyperglycemia.

Methamphetamine use results in excessive dopamine levels in the brain via D1 receptors [2], secondary to lipid solubility and ability to diffuse into the brain through the blood brain barrier [3]. Methamphetamine directly increases dopamine, reduces reuptake and inhibits monoamines which leads to prolonged extracellular dopamine activity in the brain [4]. This potency and action on the central nervous system can lead to hyperkinetic effects and psychosis [5]. Methamphetamine can also result in neurotoxicity that can lead to irreversible effects seen in imaging and brain functionality including “lower cortical gray matter density/volume consistent with reported structural abnormality in amphetamine users” [5].

Methamphetamine has been shown to result in choreoathetosis (involuntary muscle spasms and writhing); however, this effect is rare with no clear guidelines for treatment [6]. With more severe cases benzodiazepines and antipsychotics can be useful; however, mainstay of treatment in methamphetamine-induced choreoathetosis involves symptomatic management of secondary complaints including body aches and pain secondary to spasm [7]. In a similar presentation of amphetamine-induced hemichorea, a 46-year-old female patient had subsequent left leg involvement, however in this case lab work including glucose was normal [4]. In our patient, there may be some component of methamphetamine impact on the patient’s initial presentation, improvement occurred with antipsychotics, insulin and symptom control for pain.

Lurasidone is a second-generation antipsychotic which can be known to cause extrapyramidal side effects more frequently than other antipsychotics [8]. These side effects include tremor, akathisia (inability to sit still), dystonia (involuntary muscle contractions), anxiety and distress. There is no clear indication that this can be exacerbated with concurrent amphetamine use [9]; however, this was not directly addressed. Latuda operates as an antagonist for dopamine D2 and serotonin 5HT receptors [9]. Akathisia and other extrapyramidal side effects including dystonia are more noted at 120mg/day [9]; however, our patient was only taking 40mg daily. Also, lurasidone also has been shown to inhibit methamphetamine-related symptoms similar to other antipsychotics [10] which would of potentially improved symptoms and causes discussed above if methamphetamine was a cause. It is unlikely with a 40mg dose and concurrent methamphetamine use that this was the primary cause of the patient’s presentation.

Unilateral Hemichorea-Hemiballism is defined as an uncontrollable, continuous, and irregular spastic movement of one side of the body, often caused by a focal lesion of the contralateral basal ganglia [11]. Hyperglycemic Hemichorea was first described by Bedwell in 1960 where he observed 53 cases. He found the average age was 71 years old with a 1.76:1 female to male ratio. He also noted that many of the patients were Asian women suspecting a genetic predisposition [12]. Our patient is female but does not match the age or ethnicity noted in previous case. Non-ketotic hemichorea is often diagnosed via imaging with basal ganglia hyper-intensity of T1-weighted magnetic resonance images (MRI) or high density on computed tomography (CT) scans [13], both of which our patient had on imaging. Other common causes of injury to the basal ganglia can be due to vascular injury (stroke), trauma, inflammatory, neurodegenerative, or other metabolic diseases.

MRI is the gold standard of care to detect a lesion in the basal ganglia. Ballism is defined as predominantly proximal higher amplitude movements while chorea is predominantly lower amplitude and both proximal and distal movements [14]. Hemichorea associated with non-ketotic hyperglycemia (HC-NH) is a rare complication of diabetes mellitus, especially in patients that do not control their diabetes properly. The pathophysiology of this disease is not well known but chorea symptoms resolve with control over the hyperglycemia and resolve while sleeping [15]. Other causes of hemichorea-hemiballism include ischemic and hemorrhagic strokes, drugs, motor neuron disease, neurodegenerative diseases, and autoimmune disorders [16].

Utilizing glucose metabolism in the brain as a marker, chronic amphetamine users have abnormal glucose metabolism in different areas of the brain, which may have an impact on overall glucose levels. Some degree of recovery in glucose metabolism abnormalities has been noted [5]. With brain imaging and the consistent presentation of uncontrolled diabetes, it is appropriate to suggest that the primary etiology was most likely uncontrolled hyperglycemia. Specifically when factoring that improvement occurred with tighter glucose control and no other medications were changed until refusal of lurasidone. It is unclear if the other suggested etiologies did not have some role to play in our patients presentation.

The most likely primary cause of our patient's presentation is uncontrolled diabetes and chronic hyperglycemia. There is reason to suggest that the confirmed methamphetamine use and antipsychotic medication may have contributed to our patients uncontrolled hyperglycemia along with medication noncompliance. However, these likely had no direct effect on our patient's presentation.

## Conclusions

Presentations of unexplained restlessness, specifically in extremities may be secondary to many causes. In our case, the most likely cause of the patient's dystonia/chorea like movements was related to her uncontrolled diabetes, recognized by elevated A1c and glucose, brain imaging and symptom improvement with tighter glucose control. This indicated Non-Ketotic Hyperglycemic Hemichorea-Hemiballismus as primary etiology. However, in patients who present with unexplained restlessness in their extremities, other etiologies should be considered. In our patient's presentation, secondary causes that may have contributed to the initial presentation included potential antipsychotic and methamphetamine side effects. It is important to factor in comprehensive lab results, brain imaging, and pertinent history for effective treatment to occur.
